# Environmentally Realistic Levels of Total Suspended Solids Damage Gill Structure and Compromise Swimming Performance in Some Freshwater Fish Species

**DOI:** 10.3390/biology15120966

**Published:** 2026-06-19

**Authors:** Xena C. Montoya, Courtney M. Smith, William Andrew Thompson, Jonathan M. Wilson, Mathilakath M. Vijayan

**Affiliations:** 1 Department of Biology, Wilfrid Laurier University, Waterloo, ON N2L 3C5, Canada; kpxen5111@gmail.com (X.C.M.); andthompson@wlu.ca (W.A.T.); 2 Department of Biological Sciences, University of Calgary, Calgary, AB T2N 1N4, Canada; courtney.smith1@ucalgary.ca

**Keywords:** *U*_crit_, fitness, metabolic rate, rainbow trout, fathead minnow, ionocytes, Na/K-ATPase

## Abstract

Total suspended solids (TSS) have been shown to damage the structural integrity of fish gills, impairing their function, including gas exchange. However, studies showing linkages between gill damage due to TSS and fish performance are limited. There is a large diversity of fish species inhabiting aquatic environments, and whether the TSS impact on gill function is similar across a range of species has yet to be explored. Here, we exposed multiple species, including salmonids (rainbow, brook and cutthroat trout) and cyprinids (fathead minnow and longnose dace) to a range of TSS concentrations (0–1000 mg L^−1^) for 4 d and assessed damages to gill structure (filament thickness, lamellae thickness, oxygen diffusion distance, lamellae length, epithelial lifting, and interlamellar distance) using confocal microscopy. All species tested showed similar gill structural damage, including thicker lamellae, longer oxygen diffusion distances, and reduced respiratory surface area, at concentrations ≥ 100 mg L^−1^ TSS. To assess whether gill damage corresponds to performance dysfunction, we tested the metabolic rate and swimming capacity of a salmonid (rainbow trout) and a cyprinid (fathead minnow) after exposure to 100 mg L^−1^ TSS using swim tunnel respirometry. Trout showed lower routine metabolic rate (RMR) and maximum metabolic rate (MMR) after TSS exposure and were unable to reach the higher swimming speeds attained by unexposed fish. Fathead minnows showed no difference in the RMR after TSS exposure, but, like trout, had a lower MMR and were unable to attain the higher swimming speed of the control fish. Both species showed a ~35% reduction in the critical swimming speed (*U*_crit_). These findings reveal that environmentally realistic TSS concentrations damage gill structure, impair fish swimming performance, and may compromise their ability to cope with energy-demanding activities, including additional biotic and abiotic stressors.

## Simple Summary

Total suspended solids (TSS) loading is a seasonal problem in our waterways and is being exacerbated by anthropogenic activities, including land use and climate change. Studies have shown that TSS exposure affects gill morphology, but most have used only a single fish species. Here, we have taken a multi-species approach (five species, including salmonids and cyprinids) to assess whether environmentally realistic concentrations of TSS damage gills in a species-specific manner. Further, we tested whether gill damage would lead to compromised metabolic capacity and swimming performance in rainbow trout and fathead minnows. Our results revealed that 100 mg L^−1^ TSS exposure for 4 days damages the gills of all species tested and compromises the metabolic capacity and swimming performance of minnows and rainbow trout. This suggests that fish exposed to TSS may be at a disadvantage in coping with additional energy-demanding activities, including stress and escaping from predators. Our results have implications for managing in-stream TSS loading to protect fish and their habitats.

## 1. Introduction

Total suspended solids (TSS) are naturally occurring particles (>2 μm) suspended in the water column but can rise in levels due to changing climates and other anthropogenic activities [1,2]. Elevated levels of TSS have been recognized as one of the major contributors to anthropogenic disturbances in aquatic environments [3,4] and pose a considerable risk for aquatic organisms, including fish. This is because the gills, the major respiratory organ for these animals, are in direct contact with water, and the TSS have the potential to cause gill damage by abrasion [5,6,7,8]. The TSS levels in the aquatic environment range from low levels of ~50 to high levels of ~700 mg L^−1^ [9].

Studies have shown a positive linear relationship between TSS exposure and gill damage [8,10,11,12,13]. This includes thickening of lamellae and epithelial lifting, which consequently increases oxygen diffusion distance [10,11,12,13,14,15,16,17]. Gill abrasion due to TSS increases mucus production, impacting the rate of oxygen uptake across the branchial epithelium [18,19,20]. It is therefore logical to predict that these structural changes in the gills may lead to reductions in the efficiency of oxygen uptake and transport to other tissues [10,14,15,16,21].

Gills are crucial as they serve as the primary site of gas exchange and are essential for metabolism and optimal swimming performance [14,22]. The capacity of fish for oxygen uptake is related to their aerobic performance, which is measured as aerobic scope (AS), the difference between maximum metabolic rate (MMR) and routine oxygen uptake rate, and indicates the capacity to aerobically power activities above routine metabolic rate (RMR) [23]. Structural changes in the gills that reduce surface area available for gas exchange can concomitantly reduce the capacity for oxygen uptake [24]. It has been proposed that TSS-induced gill damage, which reduces surface area, can compromise the efficiency of oxygen uptake [10,11,13,14,15,16]. For instance, juvenile *Amphiprion melanopus* exposed to elevated TSS levels exhibited reduced capacity for oxygen uptake as shown by a reduction in MMR [13], but whether this is species-specific and may impact metabolic performance is far from clear [12].

The gill plays a key role in gas exchange, nitrogenous waste excretion, and ion and acid-base regulation [22]. In freshwater fish species, gill ionocytes are located in the branchial epithelium, housing ion transporters involved in moving ions and acid-base equivalents necessary to maintain homeostasis [25,26,27]. In hypoosmotic conditions in fresh water, there is a tendency for water influx across the gills and a net loss of ions via passive diffusion and ionic gradients [22,28], demonstrating the importance of transporters in maintaining osmoregulation [22]. The key drivers of ion transport and acid-base regulation include the Na^+^/K^+^-ATPase (NKA) and vacuolar type proton pump H^+^-ATPase (VHA) [29]. These active transporters, located in ionocytes, are responsible for transporting Na^+^ ions across the apical membrane, creating favorable electrochemical gradients to drive H^+^ uptake [30,31]. Although limited, previous studies have shown that elevated levels of TSS can perturb ionocytes in fish [10,15,32], suggesting that the impact of suspended solids may also be reflected in osmoregulatory disturbances. Consequently, the TSS impact on gill function may lead to a metabolic cost associated with performance dysfunction, but this remains to be determined.

To this end, the present study investigates whether (i) the TSS-induced gill damage is species-nonspecific and (ii) perturbations to gill structure due to TSS affect metabolic rate and swimming performance of fishes. We used confocal microscopy to assess gill morphological parameters indicative of gill damage and swim tunnel respirometry to measure *ṀO*_2_ (RMR, MMR, and AS) and critical swimming speed (*U*_crit_). The experimental design consisted of a multispecies comparison, including rainbow trout (*Oncorhynchus mykiss*), brook trout (*Salvelinus fontinalis*), cutthroat trout (*Oncorhynchus clarkii*), fathead minnow (*Pimephales promelas*), and longnose dace (*Rhinichthys cataractae*), of gill structural impact after a 4 d exposure to a range of TSS concentrations (0–1000 mg L^−1^). Furthermore, we compared the TSS impact on metabolic rate and swimming performance by subjecting a salmonid (rainbow trout) and a cyprinid (fathead minnow) to swim tunnel respirometry. Our results indicate that TSS at environmentally realistic levels damages fish gills and reduces their capacity for metabolic and swimming performances. This performance dysfunction may be more apparent when the animal is subjected to energy-demanding activities, including escaping predators or coping with rising temperatures. Unraveling the potential link between gill damage and fish aerobic and swimming performance may be an important marker in determining future impacts, including changes in fish populations due to TSS exposure and climate change.

## 2. Materials and Methods

### 2.1. Fish and Experimental Protocol

Two groups of freshwater fish species native to Canada, salmonids (rainbow trout, cutthroat trout, and brook trout) and cyprinids (longnose dace and fathead minnows), were used to determine the species-specific gill responses to elevated TSS exposure. Salmonids were sourced from Allison Creek Brood Trout Hatchery, Crowsnest Pass, AB, Canada [(Length in mm (L) and Weight in g (W): Cutthroat trout: (L) 73 ± 1 and (W) 3.9 ± 0.2; Brook Trout: (L) 81 ± 1 and (W) 5.3 ± 0.2; Rainbow Trout (L) 92 ± 1 and (W) 8.3 ± 0.3]. Fathead minnows were reared from the laboratory and a gift from Dr. Wiseman, University of Lethbridge, Lethbridge, AB, Canada [(L) 53 ± 0.7 and (W) 1.95 ± 0.07], while longnose dace were obtained by electrofishing from the Bow River, Calgary, AB, Canada [(L) 51 ± 1 mm and (W) 1.14 ± 0.04]. All species were acclimated for 10–14 d at the animal care facility in the Department of Biological Sciences, University of Calgary, prior to the start of the experiment. Fish were maintained in dechlorinated city water in well-aerated tanks (pH 7.5 ± 0.2) at an appropriate volume and density. All experiments were approved by the University of Calgary’s Animal Care Committee (AC21-0065) and were done in accordance with the Canadian Council of Animal Care guidelines. All fish were juveniles, so the sex ratio was not considered. The fish were fed either daily (cyprinids) or every second day (salmonids) with Bio Oregon crumb #2 trout feed. Longnose dace were caught under license 23-0215 FR and housed at the University under license 23-0117 FR, while all other species were housed under license 23-0108 FR.

The exposures were conducted in 10 L tanks with an in-house built aeration system as described previously [8]. All fish were acclimated to 10–14 d prior to the start of TSS exposure to adjust to laboratory conditions. Each tank had four juvenile salmonids or longnose dace, whereas six fathead minnows were held in each tank. Water parameters for the salmonids were kept at 10 °C, while fathead minnows were maintained at 25 °C and longnose dace at 20 °C (to reflect the temperature at capture). Water changes were performed daily (50% renewal) during the acclimation period. Levels of pH, nitrates, nitrites, and ammonia were monitored throughout and kept within safe limits. The system water was exchanged for Bow River water 48 h prior to the addition of sediments (CLNSED2-250 g; Sigma Aldrich, St. Louis, MO, USA), and this was used for all subsequent 50% water exchanges for the remainder of the exposure. Given the difficulty in obtaining the volume of Bow River water required to perform the in-lab exposures, only a 48 h acclimation period with river water was possible prior to the TSS exposures. This timing was consistent across all species tested. Fish were exposed to TSS concentrations of 0, 50, 100, 500, or 1000 mg L^−1^ in sediments for four days, with each concentration in 3 replicate tanks as described previously [8]. A second experiment was carried out to specifically examine the impacts of TSS on aerobic and swimming performance in rainbow trout and fathead minnows at 100 mg L^−1^ TSS. This concentration was selected based on gill-damage results from this study and a previous study [8]. The exposure was exactly as described above, and each tank (8 L) contained three fish (two tanks for the control and two tanks for TSS). All fish from the replicate tanks were sampled and used for the analyses.

### 2.2. Gill Histology

Following a 4-day TSS exposure, fish were euthanized with an overdose of anesthetic (MS222 1:5000; Syndel, Nanaimo, BC, Canada; buffered with 1:10,000 sodium bicarbonate) for gill tissue dissection. Left gill arches were excised with the first gill arch immersion fixed in 4% paraformaldehyde (PFA) buffered solution overnight for whole-mount confocal microscopy, and the remaining gill arches in the left basket were fixed for paraffin embedding. Fixed gills were stored in 70% ethanol prior to histological analysis.

#### 2.2.1. Whole-Mount Gill Staining

The first gill arch from the left gill basket was placed in a well of a 96-well plate containing 70% ethanol, rehydrated with MilliQ water, and rinsed with phosphate-buffered saline (PBS mM; 10.14 Na_2_HPO_4_; 1.76 KH_2_PO_4_, 2.68 KCl, 136.89 NaCl, pH 7.4). Gills were stained by incubation with 50 U/mL of CF488 Phalloidin dye (Biotium, Fremont, CA, USA) at 37 °C in the dark for 1 h. Gills were rinsed and mounted with 1:1 PBG: Glycerol mounting media with 0.1% sodium azide in a depression slide and stored at 4 °C until imaging. Slides were viewed, and images were captured using an inverted confocal laser scanning microscope (Olympus Fluoview FV1000, Tokyo, Japan) with the Olympus software (Olympus FluoView^TM^ FV1000 3.0, Tokyo, Japan). The 488 nm laser was used to observe phalloidin actin staining in gill filaments and lamellae. Gill morphometrics, including lamellae thickness (LT), filament thickness (FT), oxygen diffusion distance (ODD), lamellae height (LH), interlamellar distance (ID), and epithelial lifting (EL), were measured using Fiji ImageJ software (ImageJ2 v2.15.x, Madison, WI, USA; [33]; see Figure S1). Measurements were carried out as described previously [16], and three filaments were studied from each fish, with three fields from each filament (for a total of 9 fields per fish).

#### 2.2.2. Gill Immunohistochemistry

Fish gills were prepared for immunohistochemistry to detect VHA and NKA. Before immunohistochemistry, gills were decalcified using formic acid-sodium citrate (FASC) overnight and processed for paraffin embedding. The sections were collected on slides coated with 2% (3-Aminopropyl) triethoxysilane (APS) (Sigma Aldrich, St. Louis, Mo, USA; A3648) in dry acetone. The slides were air-dried, dewaxed, and rehydrated. The sections were blocked using BLØK (MilliporeSigma, Burlington, MA, USA) in TPBS (PBS with 0.05% Tween 20) with 5% Normal Goat Serum (NGS). A solution of 1% SDS in PBS was used for antigen retrieval [34]. Immunohistochemical staining was performed using primary antibodies against two key ion transporters in the gills, the NKA α subunit (αR1 mouse monoclonal antibody) and VHA A subunit (mouse monoclonal antibody; clone 4F5 Santa Cruz Biotech, Dallas, TX, USA). Both antibodies exhibit broad species cross-reactivity, since they recognize conserved epitopes in vertebrates (e.g., NKA α: [35,36,37] VHA-A: [38,39]). Primary antibodies were diluted to 1:100 in 1% BSA, 0.05% sodium azide in TPBS and were incubated at 37 °C for 2 h. NKA and VHA were used in pairs for double immunolabelling, and secondary goat anti-rabbit Alexa 488 and goat anti-mouse Alexa 555 antibodies were diluted 1:500 in TPBS for 2 h incubation at 37 °C. Sections were rinsed with TPBS and counterstained with DAPI (4′,6-Diamidine-20-phenylindole dihydrochloride) and were imaged using a Leica DM5500B photomicroscope; Leica Microsystems, Wetzlar, Germany) with LASX software (version 5.3.3, Leica Microsystems, Wetzlar, Germany). Images that were immunolabelled were analyzed using SigmaScanPro 5 software (SPSS; Systat Software, San Jose, CA, USA) for cell quantification of average fluorescence (red or green) intensity, ionocyte shape, and density, as described previously [40].

### 2.3. Respirometry

Rainbow trout and fathead minnows were used as the representative fish species from the salmonid and cyprinid groups to measure *ṀO*_2_ and *U*_crit_. Intermittent respirometry and *U*_crit_ measurements were performed using Brett-type (~5 L) and Blazka-type (~0.17 L) swimming respirometers with a DAQ-M controller and Autoresp2 software version 2.2 (Loligo systems, Tjele, Denmark) for rainbow trout (10 °C) and fathead minnows (25 °C), respectively. After the 96 h exposure to TSS, six fish from each group (0 vs. 100 mg L^−1^; rainbow trout 10.42 g ± 3.10 g and fathead minnow 2.13 g ± 0.56 g) were used to measure *U*_crit_ and *ṀO*_2_.

Trout swam at 5 cm/s for 1 h to obtain RMR. During the acclimation period, *ṀO*_2_ was measured in 10 min cycles, resulting in a total *ṀO*_2_ measurement of six throughout the acclimation period. To measure *U*_crit_, ramp-*U*_crit_ protocols were performed as described by Jain et al. [41]. After the acclimation period, the velocity was ramped up to 30 cm/s (50% of rainbow trout’s estimated *U*_crit_ at 10 °C) [42,43] using 5 cm/s increments every minute [44]. Thereafter, velocity increments of 10 cm/s were applied every 20 min until fish exhaustion.

FHM swam at 4.5 cm/s for 30 min to obtain the RMR. During the acclimation period, *ṀO*_2_ was measured in 5 min cycles, which resulted in a total *ṀO*_2_ measurement of 12 throughout the acclimation period. To measure *U*_crit_, a swim protocol was performed as described by Gilbert et al. [45]. After the acclimation period, *U*_crit_ was measured by which water velocity was increased gradually using 4.5 cm/s increments every 10 min until fish exhaustion. Exhaustion was defined as the point at which the fish was forced to the back of the swim tunnel, causing the fish to stop swimming for more than 5 s.

If fish were reluctant to swim during the first minute of the increment, a stimulus to initiate swimming was attempted by briefly reversing the water flow by turning the knob of the velocity control box towards reverse, then back to forward within three seconds (FIU.edu swim performance protocol; [46]). If this stimulation did not elicit swimming and the fish remained at rest at the back of the tunnel, the experiment was terminated. For the rest of each increment, exhaustion was designated when the fish was pinned and rested its tail at the back comb of the tunnel and did not regain activity after three attempts at stimulation [46]. The duration (T, in s) at the final swim speed (Vf, in cm s^−1^) was recorded, and the *U*_crit_ (cm s^−1^) was calculated using the equation described by Brett [47]:

*U*_crit_ = [Vf + (T/t)*dV*]/cm (1)

where t is the time interval, and *dV* is the increment in swim speed. Upon completion of the *U*_crit_ test, total body length (cm) was measured to convert *U*_crit_ values to body lengths per second (BL s^−1^).

Aerobic capacity was also assessed by measuring RMR and MMR. The highest oxygen consumption rate during the *U*_crit_ test was taken as the MMR, while the RMR was estimated by extrapolating all recorded *ṀO*_2_ values back to zero swimming speed [48].

### 2.4. Statistical Analysis

Data was analyzed for homogeneity of variance and normal distribution, and transformed when necessary, using the SPSS program SigmaPlot 11. A one-way ANOVA was used to determine the effects of TSS concentration on the different gill parameters, including lamella thickness, lamellae height, filament thickness, oxygen diffusion distance, epithelial lifting, and interlamellar distance. For a significant result, a post hoc (Student–Newman–Keuls) test was performed. A *t*-test was performed to compare swimming and aerobic performance (RMR, MMR, and *U*_crit_) between 0 and 100 mg L^−1^ TSS in fathead minnows. Some rainbow trout did not respond to the increase in swimming speeds and were excluded from statistical analysis due to the limited sample size (n = 1). However, the data points are still included in the figures. A two-way ANOVA was used to analyze the effect of TSS on the oxygen consumption rate (*ṀO*_2_) at different swimming speeds, followed by a post hoc Student–Newman–Keuls test.

## 3. Results

### 3.1. Gill Morphometrics

**Figure 1:**
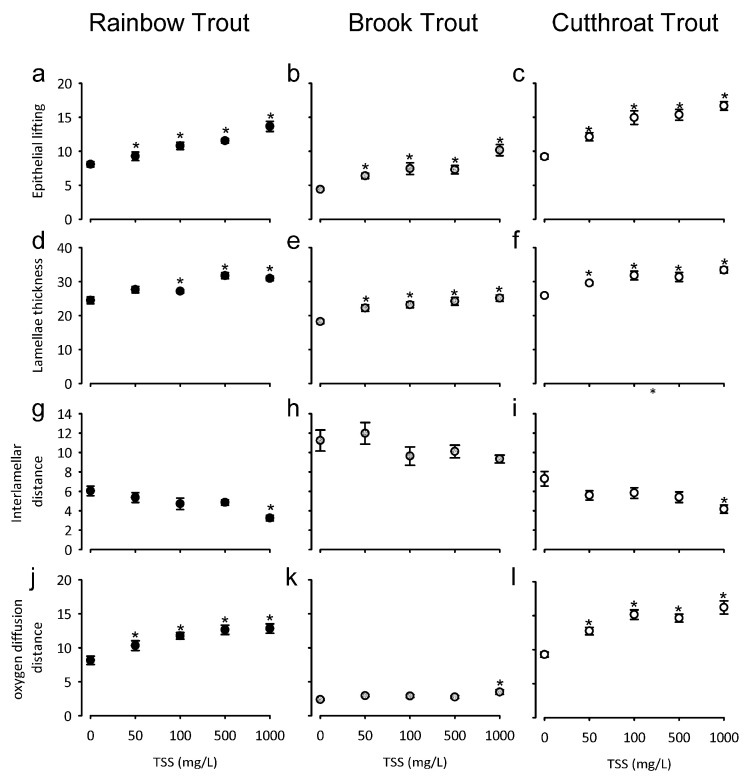
**TSS effects on the gill structure of salmonids.** Representative graphs of gill morphometric changes, including (**a**–**c**) epithelial lifting, (**d**–**f**) lamellar thickness, (**g**–**i**) interlamellar distance, and (**j**–**l**) oxygen diffusion distance, of salmonids exposed to 0, 50, 100, 500, and 1000 mg L^−1^ TSS. Black circle denotes data for rainbow trout (**a**,**d**,**g**,**j**); gray circle denotes data for brook trout (**b**,**e**,**h**,**k**); and white circle denotes data for cutthroat trout (**c**,**f**,**i**,**l**). The asterisks indicate a significant difference within a species from the control TSS (0 mg L^−1^) (one-way ANOVA, *p* < 0.05).

**Figure 2:**
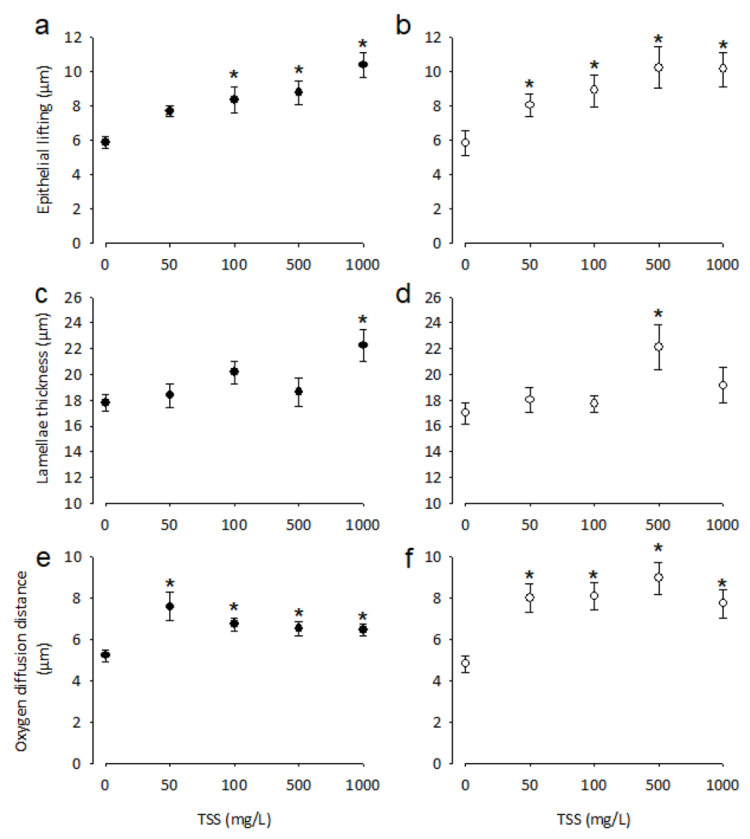
**TSS effects on the gill structure of cyprinids.** Representative graphs of gill morphometric changes, including (**a**,**b**) epithelial lifting, (**c**,**d**) lamellar thickness, and (**e**,**f**) oxygen diffusion distance in fathead minnow [FHM; (**a**,**c**,**e**)] and longnose dace [LND; (**b**,**d**,**f**)] exposed to 0, 50, 100, 500, and 1000 mg L^−1^ TSS. The asterisks indicate a significant difference within a species from the control TSS (0 mg L^−1^) (one-way ANOVA, *p* < 0.05).

Exposure to TSS significantly altered gill structure in the five fish species tested, with species-specific effects noted (see Figure S2 for representative confocal images). It was evident that gill lamellae were significantly thicker following exposure to TSS. In all three salmonid species tested, the gill lamellae were significantly thicker (up to 1.3- and 1.4-fold) at TSS concentrations ≥ 100 mg L^−1^ when compared to control fish (Figure 1, *p* < 0.05). For the fathead minnows (FHMs), gill lamellae were significantly thicker at TSS ≥ 1000 mg L^−1^ for up to 1.2-fold in comparison to the control fish (Figure 2; *p* = 0.015). Lamellar thickness in longnose dace was 1.5-fold greater in fish exposed to 500 mg L^−1^ than in gills from control fish (Figure 2; *p* = 0.034). Despite the changes observed in the lamellar thickness in all species, interlamellar distance was only significantly lower in two species, the rainbow trout and cutthroat trout, at 1000 mg L^−1^ TSS (Figure 1; RT: *p* ≤ 0.001; CT: *p* = 0.006).

The gill lamellae of all five species exposed to TSS exhibited significantly higher oxygen diffusion distance when compared to the control fish. In all species, the oxygen diffusion distance was significantly longer at all TSS concentrations as low as 50 mg L^−1^ (Figure 1 and Figure 2; *p* ≤ 0.001) except for brook trout, which was significantly longer only at 1000 mg L^−1^ (Figure 1; *p* = 0.017). Gill damage via its abrasive capabilities was evidently shown by the high degree of epithelial lifting in all fish species exposed to TSS concentration ≥ 50 mg L^−1^ (Figure 1; *p* ≤ 0.001 and Figure 2; *p* ≤ 0.001).

### 3.2. Swimming Respirometry Studies: ṀO_2_ and U_crit_

**Figure 3:**
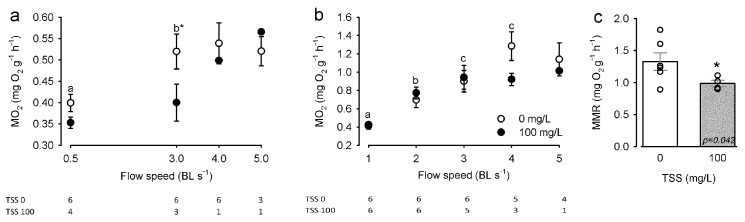
**TSS effects on metabolic rate.** The oxygen consumption rate (*ṀO*_2_) during the swim test at different flow speeds in (**a**) rainbow trout and (**b**) fathead minnows exposed to 0 and 100 mg L^−1^ TSS is shown. The number of fish that completed the swimming trial at the flow speed indicated above is shown in a table below the plots. Flow speed with different letters is significantly different (flow speed effect), while an asterisk indicates a significant difference between the treatments within a flow speed (interaction, two-way ANOVA, *p* < 0.05). (**c**) The maximum metabolic rate (MMR) in fathead minnows exposed to 0 and 100 mg L^−1^ of TSS; an asterisk denotes a significant difference from control fish (Student’s *t*-test, *p* < 0.05).

The *ṀO*_2_ was significantly lower (12%) in rainbow trout exposed to 100 mg L^−1^ in comparison to the control fish (Figure 3a). The *ṀO*_2_ in fathead minnows exposed to 100 mg L^−1^ was not different in comparison to the unexposed control fish (Figure 3b). However, the measured MMR was significantly lower in fathead minnows exposed to 100 mg L^−1^ TSS by 20% (Figure 3c). RMR (0 mg/L: 1.51 ± 0.47 mg O_2_ g^−1^ h^−1^; 100 mg/L: 2.19 ± 0.98 mg O_2_ g^−1^ h^−1^) and AS (0 mg/L: 11.78 ± 1.72 mg O_2_ g^−1^ h^−1^; 100 mg/L: 7.66 ± 1.45 mg O_2_ g^−1^ h^−1^) did not show differences between control and TSS groups in fathead minnows. Trout also showed a reduction in MMR at 30 cm s^−1^.

**Figure 4:**
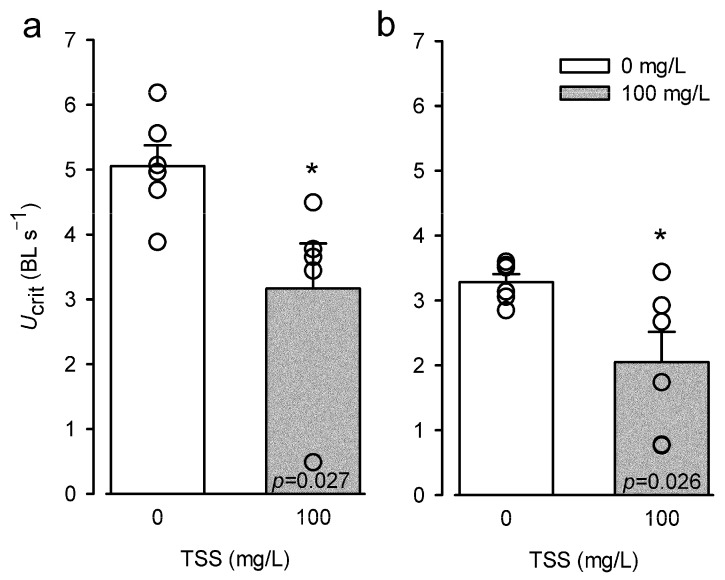
**TSS effects on critical swimming speed.** Mean critical swimming speed (*U*_crit_) in (**a**) rainbow trout and (**b**) fathead minnows following exposure to 0 and 100 mg L^−1^ TSS for 96 h (n = 5–6). Fish were swum individually in either a Brett-type (rainbow trout) or a Blazka-type swimming respirometer (fathead minnow). An asterisk denotes a significant difference from control fish (Student’s *t*-test, *p* < 0.05).

The critical swimming speed of representative species from each group, salmonids and cyprinids, was measured to determine the impacts of TSS exposure (100 mg L^−1^) on fish swimming performance. The mean *U*_crit_ of unexposed rainbow trout was 5.06 ± 0.319 BL s^−1^, while it was 3.28 ± 0.14 BL s^−1^ for fathead minnows. Exposure to 100 mg L^−1^ revealed a significant reduction in rainbow trout *U*_crit_ by ~37% (Figure 4a; 3.17 ± 0.693 BL s^−1^; *p* = 0.027). For fathead minnows, there was also a significant reduction in *U*_crit_ by ~38% (Figure 4b; 2.05 ± 0.44 BL s^−1^; *p* = 0.026).

### 3.3. Immunohistochemistry

**Figure 5:**
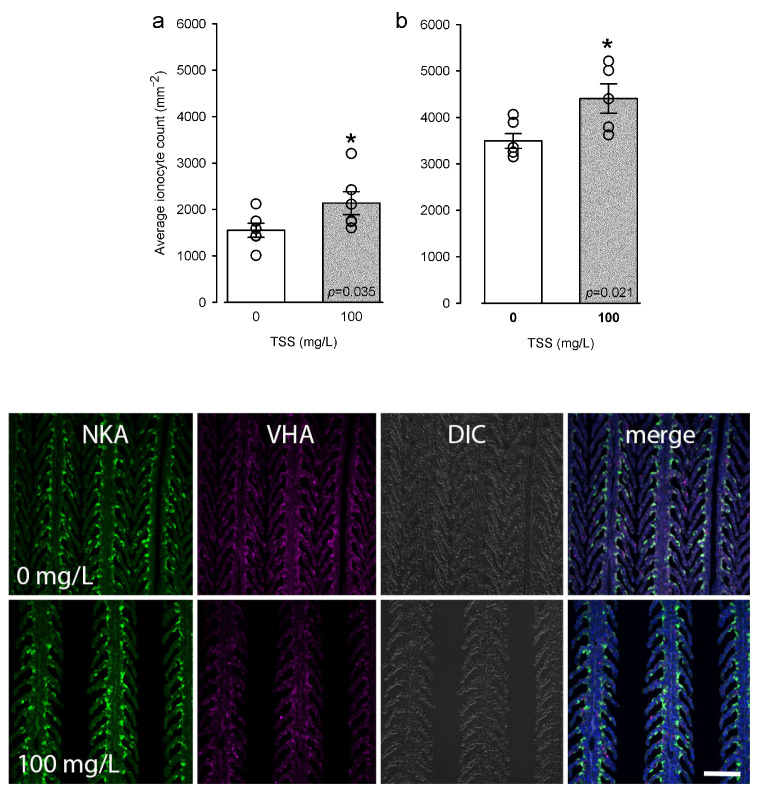
**TSS effects on the gill ionocyte count.** Average ionocyte count for Na^+^/K^+^-ATPase α-subunit (NKA) in (**a**) rainbow trout and (**b**) fathead minnow exposed to 0 and 100 mg L^−1^ TSS. Representative images of immunolabelled rainbow trout gill ionocyte for NKA (green) and vacuolar type proton pump H^+^-ATPase (VHA; magenta) at 0 mg/L and 100 mg L^−1^ TSS. Corresponding DIC (differential interference contrast) images shown with the merged fluorescence channels with DAPI (blue; nuclear stain). Scale bar 100 µm. An asterisk denotes a significant difference from control fish (Student’s *t*-test, *p* < 0.05).

The cell size, shape, and average fluorescence intensity in gill ionocytes immunolabelled with NKA and VHA were not significantly affected following exposure to 100 mg L^−1^ of TSS in the rainbow trout and fathead minnow. However, the average ionocyte count was significantly higher in both species when exposed to 100 mg L^−1^ TSS (Figure 5a,b). Exposure to 100 mg L^−1^ of TSS increased the NKA ionocyte density by 37.8% (*p* = 0.035) in rainbow trout and by 26.2% (*p* = 0.021) in fathead minnow.

## 4. Discussion

Changes in climate and anthropogenic activities increase levels of TSS in aquatic environments, which can have detrimental effects on aquatic ecosystems [1,2]. Gill structural changes associated with elevated levels of TSS have been shown to reduce the respiratory surface area, which can diminish metabolic performance due to limited capacity for efficient oxygen uptake and transport [10,11,14,15,16,21]. In the present study, we demonstrate that gill structural changes are a generalized response to TSS in fish, in agreement with previous reports, and may be an adaptive or compensatory response [8,10,11,12,15,49]. The gill damage observed with TSS corresponded with metabolic rate changes and compromised swimming performance in rainbow trout and fathead minnows, suggesting a reduced capacity for aerobic metabolic performance. In the aquatic environment, TSS-mediated reductions in aerobic capacity may limit the fish’s capacity for other energetic activities, including foraging, escaping predators, and adaptations to secondary stressors, thereby reducing animal fitness.

While TSS can naturally occur in aquatic environments, increasing levels and duration of exposure can vary spatially and temporally, and seasonally due to changes in water flow (2). The TSS levels in water bodies usually range from a low of ~50 mg L^−1^ up to 700 mg L^−1^ [5,6,7]. Consequently, our results indicate that exposure to TSS for 4 days at levels as low as 100 mg L^−1^, which is environmentally realistic, is sufficient to impact fish gill structure and function. This concentration resulted in gill damage, including the thickening of gill lamellae, an increased degree of epithelial lifting, and an increasing oxygen diffusion distance, all of which may contribute to reduced oxygen uptake [50]. Indeed, these gill morphological changes in trout and fathead minnow corresponded to impaired metabolic and swimming performance, indicating a reduced fitness for energy-demanding activities, including coping with additional stressors.

Alterations to gill morphology have not always equated to disturbances in aerobic metabolism. For instance, higher turbidity (~26.6 mg L^−1^) increases the gill lamellar oxygen diffusion distance, but without impairing whole-animal oxygen consumption rates in the Australasian snapper (*Pargus auratus*; [12]). Given that the Australasian snapper inhabit coastal waters, which are often subjected to more turbid conditions [11], it was proposed that the impaired aerobic metabolism may require higher TSS levels in this species [12]. However, in a coral reef species, the cinnamon clownfish (*Amphiprion melanopus*), exposure to increasing levels of TSS reduced the diffusion distance of oxygen, which coincided with an elevation in oxygen consumption rate [13]. Together, these results suggest that a species-specific response to TSS may vary across habitats, including chronic exposure to sediments. In our study, which involved freshwater fish species that were predominantly hatchery-reared, short-term exposure to TSS damaged gill structure and resulted in compromised swimming performance. The longnose dace was the only species caught from the wild for this study, and it also showed similar gill structural damage to TSS as the other four species. The impact may not be evident under resting conditions but may become apparent when challenged with energy-demanding activities, including physiological responses to abiotic stressors imposed by climate change. It is also possible that alternative histopathological analysis approaches may have yielded greater sensitivity, but we are confident in our comparative analysis using the parameters outlined as described previously [8,16]. We also did not assess the potentially confounding effects of bacterial or parasitic infections of the gills, but the TSS exposures were relatively short in duration, and the TSS was of commercial origin.

Critical swimming speed is an important biomarker of fish health, serving as an indicator that a fish has been exhausted aerobically and anaerobically [51]. It is clear in the present study that exposure to environmental levels of TSS (100 mg L^−1^) has the capacity to reduce the swimming performance of fish. This may be associated with reduced oxygen uptake capacity due to gill damage. Indeed, both rainbow trout and fathead minnow exhibit a reduced metabolic capacity, with rainbow trout failing to increase their oxygen consumption when swum at 3 BL s^−1^, and fathead minnows exhibiting a diminished MMR. The reduction in MMR, a measure of metabolism that reflects the maximal rate of oxygen delivery to tissue mitochondria [48], may imply a reduced capacity for oxygen uptake at the gill. Interestingly, the routine *ṀO*_2_ between the TSS-exposed and control minnows was not different, despite damage to the gills. This, however, did not occur in scenarios of elevated activity, as the MMR of minnows was reduced in the TSS group, hinting at an effect on the aerobic capacity of these animals. Aerobic scope represents the capacity for oxygen delivery beyond what is required for basal energy expenditure [48]. The aerobic capacity of fish has been linked to fitness and performance [52,53,54], and TSS-mediated reductions in oxygen uptake under strenuous activity may suggest limitations in the survivability of wild fish populations with increasing TSS levels.

The results of this study suggest that TSS exposure may also lead to disruptions in ionoregulation, particularly given the significant increase in ionocyte density observed in trout and fathead minnows. In response to osmotic stress or changing ionic gradients, ionocyte density increases (reviewed by [55]), which is necessary to maintain the ionic flux required for osmoregulation. For example, in milkfish (*Chanos chanos*), the upregulation of Na^+^/K^+^ ATPase occurs following transfer from seawater to freshwater, aiding in the maintenance of plasma ions [56]. Previous studies have also shown ionocyte changes in response to TSS, but the results are far from conclusive. For instance, TSS increased ionocyte density in green grouper (*Epinephelus coioides*) [10], but this was not the case in pond-raised catfish (*Pangasianodon hypophthalamus*; [17]) and three species of darter [57]. While this may have initially suggested that saltwater fish were more responsive to TSS exposure, the results presented herein, with ionocyte numbers increasing in both rainbow trout and fathead minnow, instead implicate possible species-specific sensitivities. We did not explicitly explore whether this change in ionocyte number was in response to a perturbation in ionoregulation, but given the increasing prevalence of TSS in the environment, follow-up studies expanding on species and osmoregulation may be prudent.

Importantly, the results here may describe a common response to high levels of TSS in freshwater fish. We note that following exposure to TSS, all five species testing within this study exhibit signs of epithelial lifting and thickening of the filaments, a common defensive mechanism in response to abrasive or toxic exposures in fish [10,58]. In response to increases in TSS, gills exhibit a similar protective action, physically altering the shape of the gill, a result seen in darters [57], *Pagrus auratus* [12], zebrafish (*Danio rerio*; [8]), and green grouper [10]. Inherently, these alterations may compromise osmoregulation and metabolism, disturbances that have been observed in this study. However, damage to gill morphology and a response such as an increase in ionocyte density may imply disturbed ionoregulation [58,59]. Although we used uncontaminated sediments for the study, TSS can also act as a vector for aquatic contaminants in the aquatic environment [60,61]. Contaminants that alter gill integrity, such as metals, may exacerbate osmoregulatory disturbances, prompting future studies to investigate the combined effects of toxicants with TSS on gill structure.

In contrast to the rainbow trout, fathead minnows were able to maintain a stable oxygen consumption rate during intense exercise (*U*_crit_ test). This may be explained by the general high tolerance displayed by fathead minnows to water quality parameters such as pH, turbidity, and temperature [62,63,64]. Rainbow trout may be more sensitive to TSS, a contention supported by strong avoidance of turbid waters by salmonids in natural settings [65,66,67]. Future studies are warranted to determine the thresholds of sensitivities of aquatic species to TSS.

## 5. Conclusions

Overall, exposure to TSS as low as 100 mg L^−1^, which is environmentally realistic, can cause gill structural damage that increases oxygen diffusion distance and therefore reduces aerobic and swimming performance in salmonid and cyprinid species. Specifically, elevated levels of TSS led to thicker gill lamellae in all five species tested due to gill epithelial lifting and longer oxygen diffusion distance. Structural changes in the gills corresponded with impacts on the oxygen uptake efficiency in rainbow trout and fathead minnow via reduction of *ṀO*_2_ and *ṀO*_2max_, respectively. TSS exposure also reduced *U*_crit,_ indicating an overall impact on swimming performance. We also found that elevated levels of TSS increase NKA ionocyte density, suggesting potential disruption in the maintenance of ion and acid-base status. Together, these results highlight a generalized effect on animal performance in response to four-day TSS exposure. Combining this study with the prevailing literature suggests that the effects of TSS on fish are species-specific; however, we have shown that rainbow trout, cutthroat trout, brook trout, longnose dace, and fathead minnows are sensitive to current TSS levels. Understanding the physiological impacts of elevated TSS levels on fish is key to determining possible outcomes should an event occur where TSS loads into aquatic systems increase. TSS levels in waterways have the potential to escalate in the future due to rising flow rates and longer flooding durations [68], and specific strategies are essential to mitigate harmful effects on aquatic species. To counteract these effects, we need to fully understand the risks posed by elevated TSS levels to fish species inhabiting our aquatic environment.

## Data Availability

The raw data supporting the conclusions of this article will be made available by the authors on request.

## Supplementary Materials

The following supporting information can be downloaded at: https://www.mdpi.com/article/10.3390/biology15120966/s1, Figure S1: Schematic of (A) first left gill arch showing two rows of gill filament protruding perpendicularly from the gill arch; (B) A cross-section through the filament showing the structure of gill lamellae; (C) schematics depicting the measurements (μm) for the different gill morphometric parameters including filament thickness (FT), lamellae height (LH), epithelial lifting (EL), interlamellar distance (ILD), oxygen-water diffusion distance (O-WD), and lamellae thickness (LT).; Figure S2: Representative confocal images with phalloidin fluorescence for morphometric analysis of gills from Rainbow trout, Brook trout, Cutthroat trout, Fathead minnow and Longnose dace. Abbreviations: F filament, L lamellae, arrowheads indicate epithelial lifting. Scale bars 50 μm.
