# Supplementary material for: Environmentally Realistic Levels of Total Suspended Solids Damage Gill Structure and Compromise Swimming Performance in Some Freshwater Fish Species
Source: Biology (Basel). 2026 Jun 19;15(12):966. doi: 10.3390/biology15120966 (PMC13296938; doi:10.3390/biology15120966)
Supplement: Supplementary file 1 [file biology-15-00966-s001.zip › biology-4258428-supplementary.pdf]

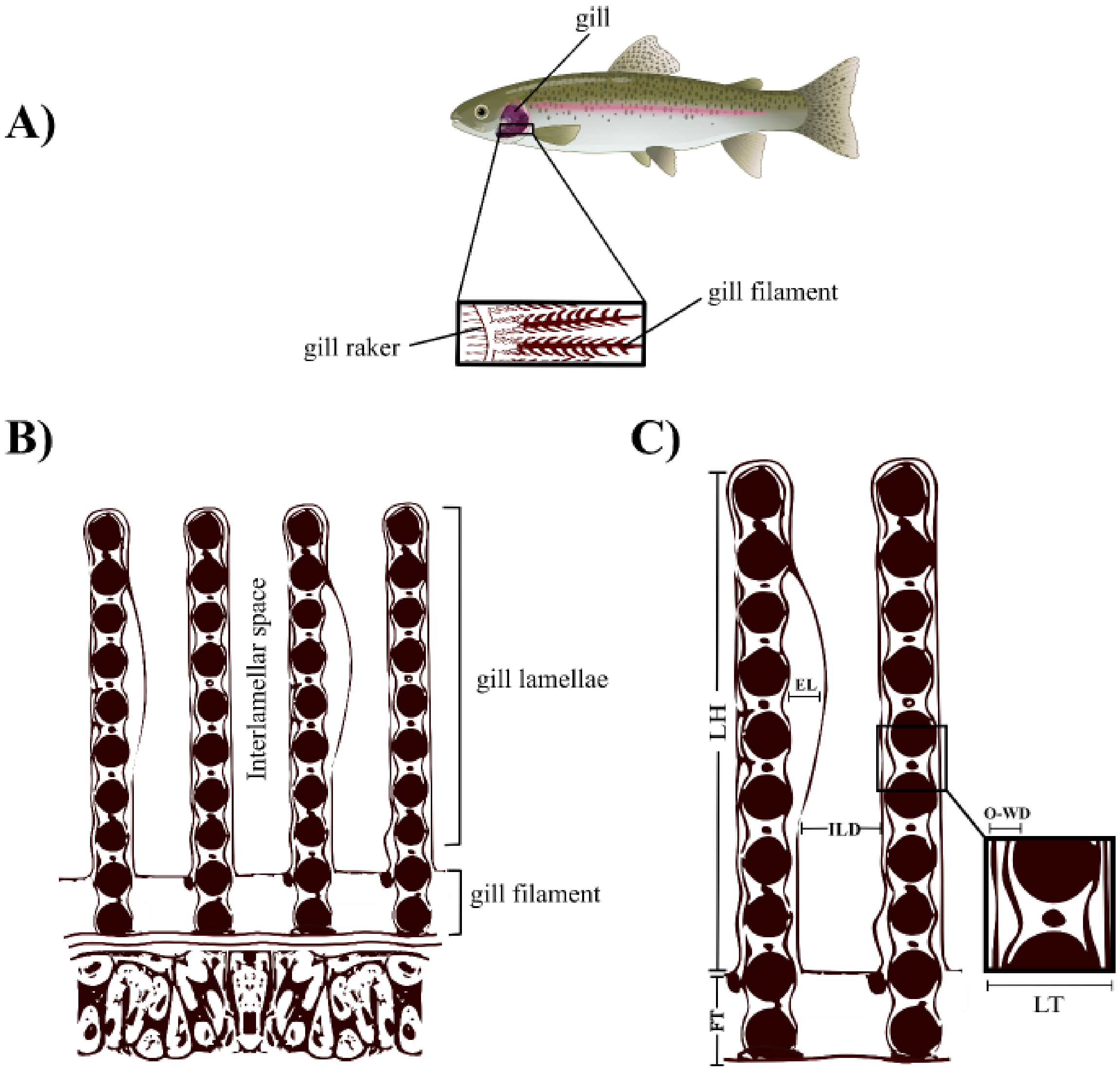

Figure S1. Schematic of **A**) first left gill arch showing two rows of gill filament protruding perpendicularly from the gill arch; **B)** A cross-section through the filament showing the structure of gill lamellae; **C)** schematics depicting the measurements (μm) for the different gill morphometric parameters including filament thickness (FT), lamellae height (LH), epithelial lifting (EL), interlamellar distance (ILD), oxygen-water diffusion distance (O-WD), and lamellae thickness (LT).

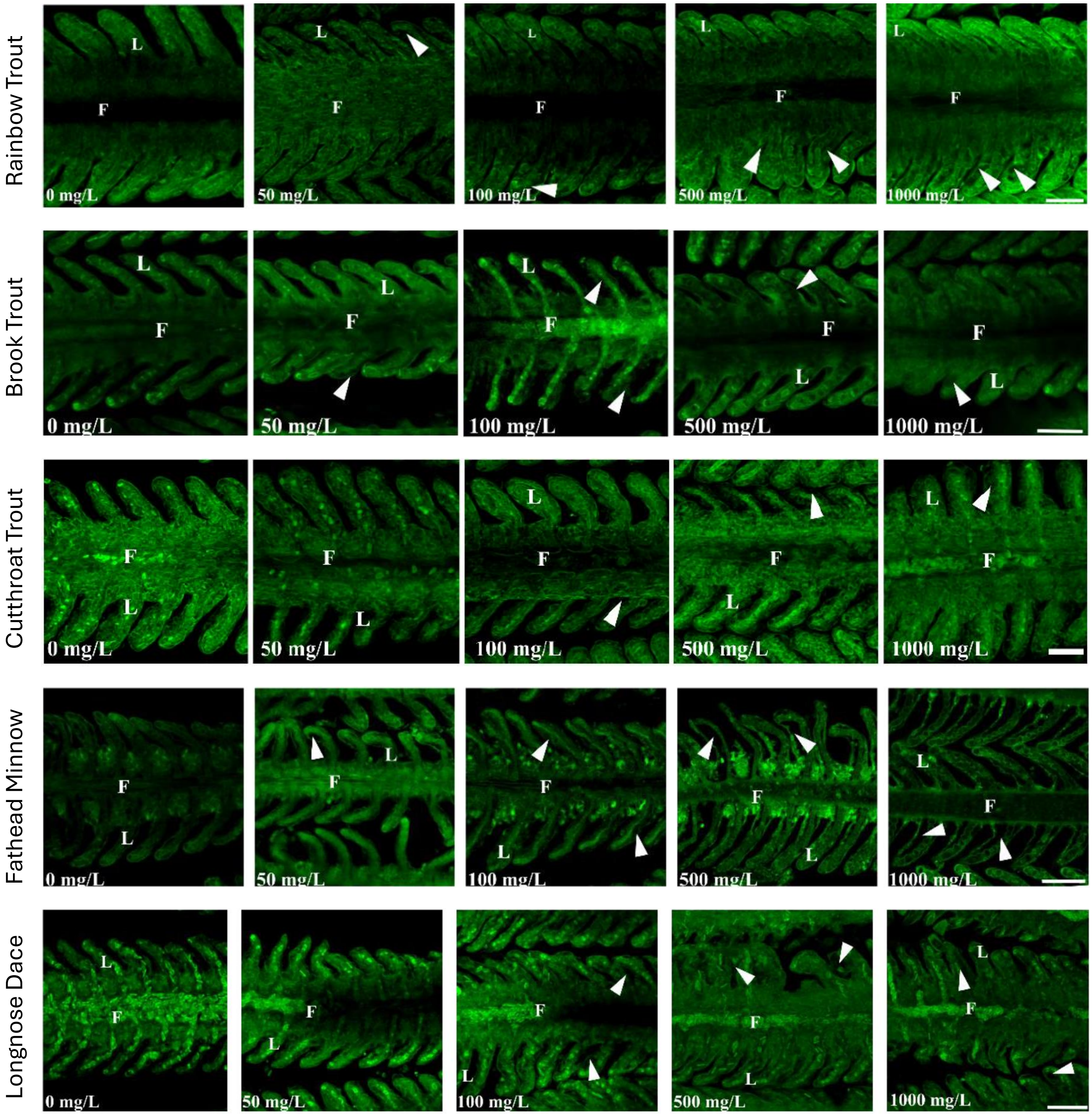

Figure S2. Representative confocal images with phalloidin fluorescence for morphometric analysis of gills from Rainbow trout, Brook trout, Cutthroat trout, Fathead minnow and Longnose dace. Abbreviations: F filament, L lamellae, arrowheads indicate epithelial lifting. Scale bars 50 μm.
